# Histogram Adjustment of Images for Improving Photogrammetric Reconstruction

**DOI:** 10.3390/s21144654

**Published:** 2021-07-07

**Authors:** Piotr Łabędź, Krzysztof Skabek, Paweł Ozimek, Mateusz Nytko

**Affiliations:** Faculty of Computer Science and Telecommunications, Cracow University of Technology, Warszawska 24, 31-155 Kraków, Poland; piotr.labedz@pk.edu.pl (P.Ł.); krzysztof.skabek@pk.edu.pl (K.S.); mateusz.nytko@pk.edu.pl (M.N.)

**Keywords:** photogrammetry, preprocessing, enhancement, point cloud, 3D reconstruction, image processing, image histogram

## Abstract

The accuracy of photogrammetric reconstruction depends largely on the acquisition conditions and on the quality of input photographs. This paper proposes methods of improving raster images that increase photogrammetric reconstruction accuracy. These methods are based on modifying color image histograms. Special emphasis was placed on the selection of channels of the RGB and CIE L*a*b* color models for further improvement of the reconstruction process. A methodology was proposed for assessing the quality of reconstruction based on premade reference models using positional statistics. The analysis of the influence of image enhancement on reconstruction was carried out for various types of objects. The proposed methods can significantly improve the quality of reconstruction. The superiority of methods based on the luminance channel of the L*a*b* model was demonstrated. Our studies indicated high efficiency of the histogram equalization method (HE), although these results were not highly distinctive for all performed tests.

## 1. Introduction

Until recently, photogrammetric reconstruction was available to a narrow community of remote sensing specialists [1,2]. It has become widely available thanks to the development of mobile applications using cameras installed in smartphones [3]. However, reconstructions made with these applications do not provide sufficient reliability for engineering applications [4]. Its potential use is building surveying, cost estimation, modeling space for virtual and augmented reality [5,6,7,8]. Unfortunately, it is very difficult to extract precise data allowing for sufficiently precise modeling from point clouds generated by these technologies [9,10,11]. This led to a search for methods to improve reconstruction quality and reliability [12,13,14]. The trust of engineers in this reconstruction is crucial [15].

Photographs are not always taken correctly. This depends on the lightning conditions [16], the availability of a given property, the amount of time one can spend on registration, the quality of the photo equipment and many other factors [17,18]. The image file format should also be taken into account when capturing images [19]. The quality of the photographs is especially important for the automated photogrammetric reconstruction process [20,21]. Its impact is particularly noticeable when processing large image datasets [22], or using the markerless method [23]. Therefore, there is a need to correct and improve the quality of these photos. Image preprocessing is used to enhance the quality of images [24] and it is a very significant issue from the point of view of 3D modeling and photogrammetric reconstruction [25]. It includes many different methods such as histogram enhancement, color balancing, denoising, deblurring or filtering [26]. Some methods have already been used to improve photo quality in the photogrammetric reconstruction pipeline. Many of these are associated with the deblurring of images [27,28,29]. The literature also includes studies on the use of using polarizing filters or High Dynamic Range (HDR) images, [25] decolorization [30] or novel techniques for the conversion of the color signal into a grayscale [31]. Significant research within this area has been reported by Gaiani et al. [32]. They developed an advanced pipeline consists of color balancing, image denoising, color-to-gray conversion and image content enrichment, including new methods for image denoising and grayscale reduction. Another result of evaluating the impact of image processing for photogrammetry were presented by Feng et al. [33]. Presently, neural networks, especially the ones based on deep models, are used to improve photogrammetric quality [29,34,35,36].

On the contrary to create the advanced photogrammetric pipeline [32] or use sophisticated tools, such as neural networks we looked for a simple way to improve photos to obtain the best material for photogrammetric reconstruction. We assumed that selected methods of improving images, based on histogram operations, should give the desired results. Our methodology is designed to work directly on photographs, as a preprocessing step in the photogrammetric reconstruction pipeline. It can be used independently of reconstruction software, which required testing with black-box type tool. We verified the correctness of our method by comparing the reconstructions obtained from the preprocessed materials with the most reliable geometric model at our disposal. In addition to methods of improving image quality, finding a good way to compare the reconstruction to a reliable model is not an obvious task.

In our previous work [37] we explored the improvement of input images for photogrammetric reconstruction. Our conclusion was that reconstruction using unprocessed photos does not always give satisfactory results. We tested several methods of image preprocessing based on histogram analysis however, we focused only on grayscale images in that paper. Reconstruction based on such images is correct in terms of structure, but it is also somewhat limited as it is not possible to use colored textures. To correct this inconvenience, we tested the following image processing methods: histogram stretching (HS), histogram equalizing (HE), adaptive histogram equalizing (AHE) and exact histogram matching (EHM) on color images with the use of different color spaces.

## 2. Materials and Methods

### 2.1. Research Method

Several methods of processing photographic material were selected for our study. The processing results were used to generate photogrammetric reconstructions of photographed objects. The reconstructions were in the form of point clouds. Each reconstruction was compared with a suitable reference model. The reference models featured a representation of polygonal meshes. The aim of our study was to characterize individual material processing methods in terms of their suitability for generating geometrically correct point clouds. Therefore, the selection of reliable reference models and reliable methods for comparing reconstructions to reference models were an important element of our study.

Two real-world objects were selected as case studies. The first was a porcelain swan figurine. The second was a large historical building. Analyses carried out on objects of such different scales resulted in a wide range of recommendations regarding the methods of processing photographic material that had been tested. At the same time, they required other methods of obtaining reference models. Unfortunately, different modeling methods give models with varying degrees of fidelity. When selecting the appropriate measures for such different objects, the same methods of comparing the reconstruction with the models were used. The results of these comparisons were also characterized in relation to the scale of the object.

### 2.2. Photo Improvement Methods

**Figure 1:**
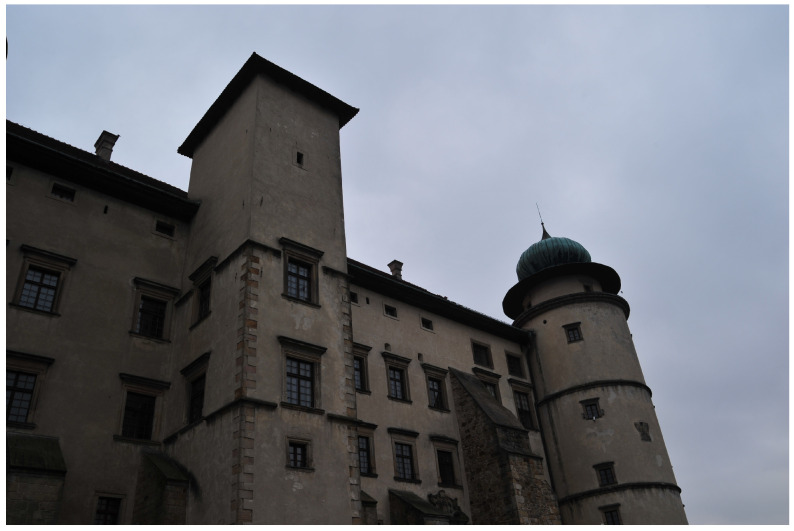
Example of an underexposed image.

**Figure 2:**
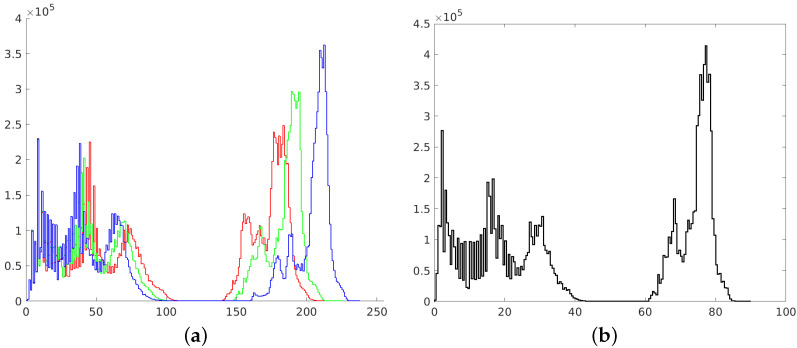
Histograms of RGB channels (**a**) and L channel of L*a*b* space (**b**).

In this paper, we focused on improving the quality of images, mainly in terms of contrast and brightness. We did not explore the influence of other factors (such as sharpness), which shall be a part of further research. We focused only on color photographs (Figure 1), as we already published research on monochrome images [37]. Two different color spaces are used in the imaging processing—RGB and L*a*b*. The RGB space image was divided into three separate channels—red, green and blue. Each channel was treated as a single monochrome image, therefore histogram corrections were performed on each channel separately (Figure 2), and the RGB image was recombined afterwards. In the L*a*b* space image was also divided into three separate channels – L*, a* and b*. The L* channel represents brightness (luminance), a* channel color position between red and green and b* channel color position between yellow and blue. In order to preserve the original colors of an image, modifications were introduced into the L* channel only. For this channel to be considered a monochrome image, its values were previously transformed to the [0 1] range.

As described in Algorithm 1 each image needs to be converted back into RGB space to perform a photogrammetric reconstruction.

#### 2.2.1. Histogram Stretching (HS)

**Figure 3:**
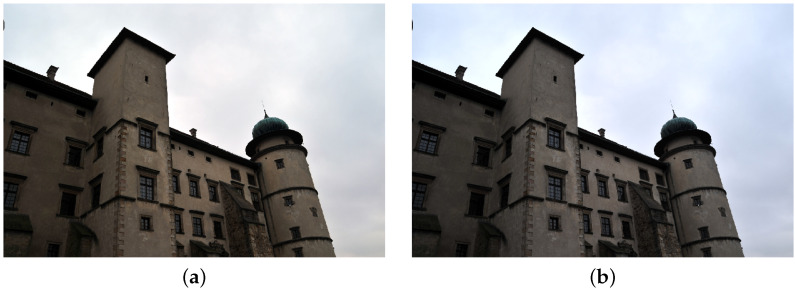
Image transformed with the use of histogram stretching in RGB (**a**) and L*a*b* space (**b**).

**Figure 4:**
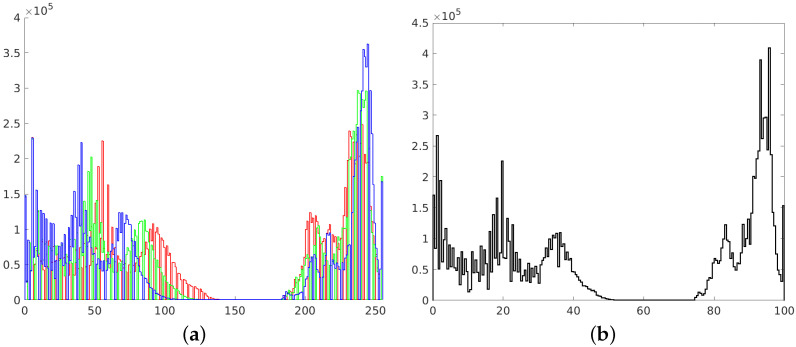
Histograms of RGB channels (**a**) and L channel of L*a*b* space (**b**).

The first image enhancement method that we used was common histogram stretching. This process maps the intensity values of the image into new values to redistribute the information of the histogram toward the extremes of a gray-level range. This increases the intensity range, although some of the brightness values are not represented in the processed image [38]. Transformed images with their histograms are presented in Figure 3 and Figure 4.
**Algorithm 1:** Histogram modification in L*a*b* space   **Input**:  $I_{RGB}$—image in RGB space
**1** convert $I_{RGB}$ into $I_{L*a*b*}$;
**2** divide $I_{L*a*b*}$ into separate channels;
**3** leave a∗ and b∗ channels without any changes;
**4** transform L∗ channel to the [0 1] range;
**5** perform certain histogram operation on L∗ channel;
**6** transform back L∗ channel to the [0 100] range;
**7** combine $I_{L*a*b{*}^{\prime }}$ from separate channels;
**8** convert $I_{L*a*b{*}^{\prime }}$ into $I_{RGB^{\prime }}$;
**9**
**return** $I_{RGB^{\prime }}$—modified image in RGB space;


#### 2.2.2. Histogram Equalizing (HE)

**Figure 5:**
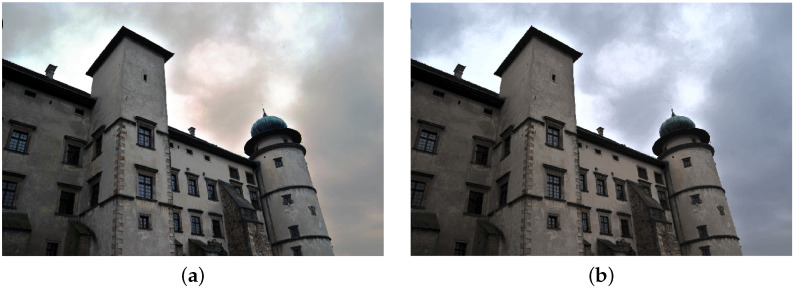
Image transformed with the use of histogram equalizing in RGB (**a**) and L*a*b* space (**b**).

The second way to improve image contrast that we tested was the histogram equalization. This technique effectively stretches the most common intensity values—it extends the intensity range of an image. Afterwards, intensities can be distributed better—low-contrast areas will have higher contrast and the cumulative histogram would increase linearly [24]. Performing histogram equalization on separate RGB channels often leads to unrealistic effects. Therefore, this operation is more suitable for images in different color spaces, such as L*a*b* (Figure 5).

**Figure 6:**
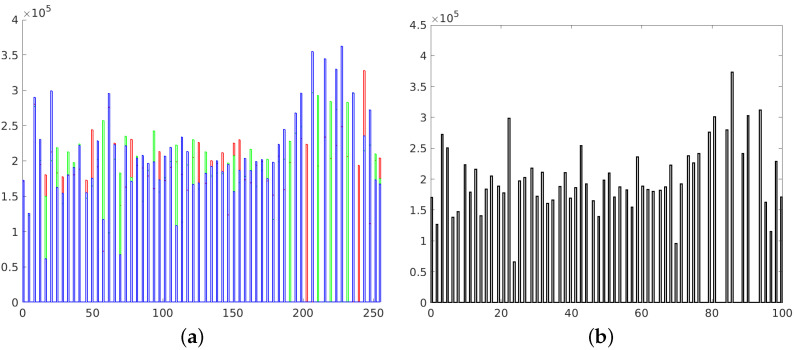
Histograms of RGB channels (**a**) and L channel of L*a*b* space (**b**).

The resulting histograms are as flat as possible, without noticeable peaks. However, it should be noticed that many intensities are not represented in obtained images. This is represented as white gaps between the histogram bars (Figure 6).

#### 2.2.3. Adaptive Histogram Equalizing (AHE)

The third method of improving images that we tried was adaptive histogram equalization. This method differs from ordinary histogram equalization in the way that the adaptive method (AHE) calculates histograms in separate parts of the image (tiles) instead of the entire image [39]. In each tile, a transformation function is calculated for each pixel based on neighboring values. The classic AHE approach tends to overamplify contrast and noise. Therefore, contrast-limited adaptive histogram equalization (CLAHE) was applied in place of AHE. In this algorithm, the histogram is first truncated at a predefined value and the transformation function is calculated afterwards. This situation takes place especially in homogeneous areas [40].

**Figure 7:**
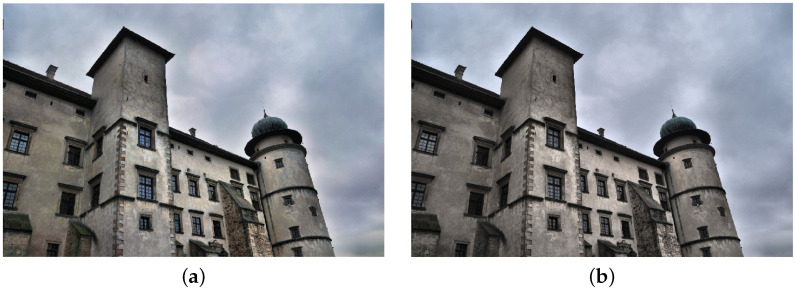
Image transformed with the use of adaptive histogram equalizing in RGB (**a**) and L*a*b* space (**b**).

**Figure 8:**
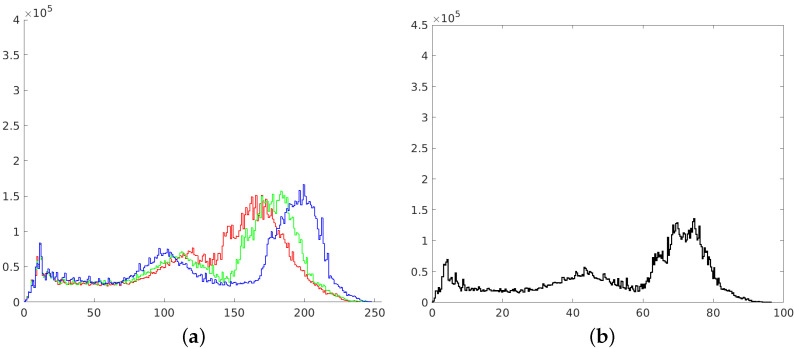
Histograms of RGB channels (**a**) and L channel of L*a*b* space (**b**).

After performing the equalization, an algorithm combines neighboring tiles using bilinear interpolation to eliminate artificially induced boundaries. The resulting image and its histogram differ significantly from the image obtained with the use of the standard histogram equalization technique (Figure 7 and Figure 8).

#### 2.2.4. Exact Histogram Matching (EHM)

**Figure 9:**
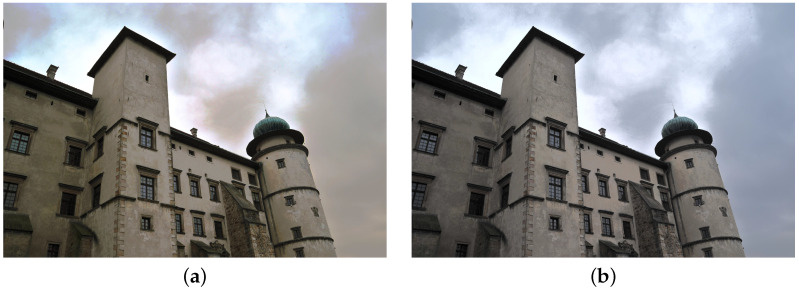
Image transformed with the use of exact histogram matching in RGB (**a**) and L*a*b* space (**b**).

**Figure 10:**
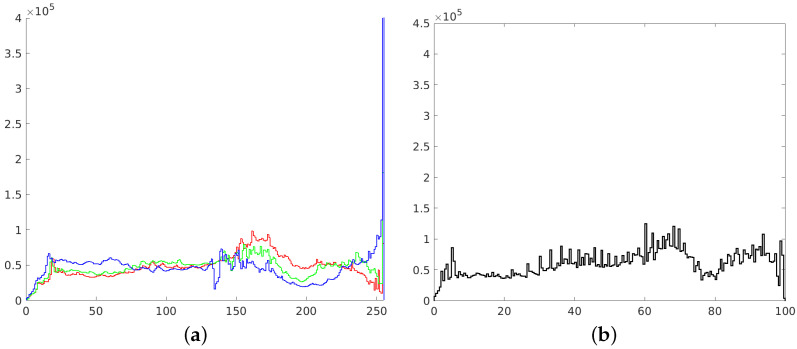
Histograms of RGB channels (**a**) and L channel of L*a*b* space (**b**).

The last image enhancement process consists of two main stages. In the first stage, a mean histogram of the whole set of images is calculated. This is performed by adding histograms of each image (separately for each channel) and dividing the result by the number of images in the set. In the second step, an exact histogram matching operation [41,42] is used to adapt the histogram of each image to match the obtained average histogram (Figure 9 and Figure 10).

### 2.3. Photogrammetric Reconstruction

For photogrammetric reconstruction, we used Agisoft Metashape, which is used extensively worldwide. During the reconstruction phase, the same parameters were used for each step to keep the test method consistent. The reconstructions were created independently for each set of photos, i.e., the original photos and those transformed following the algorithms described. We chose two types of objects to validate the method’s correctness regardless of the scale of the object subjected to photogrammetric reconstruction. Comparisons between individual reconstructions were performed using Cloud Compare software.

Agisoft Metashape provides a built-in tool for assessing the quality of photos, but this is based on sharpness only. For this purpose, it analyzes contrast between pixels and determines the quality factor which takes a value from 0 to 1. According to the manufacturer’s recommendation, images with a factor lower than 0.5 should be excluded from the reconstruction process. This criterion is not always reliable, because in the case of directional blur, which is most common when taking pictures, sharp areas can still be detected by algorithm, and qualify the picture as good quality [43]. There is no information about any image preprocessing techniques implemented in Metashape, therefore it should be treated as a black-box tool.

A DSLR camera was used in the registration of photographs for the photogrammetric reconstruction. The sensor was not calibrated in any of the described case studies, in addition a markerless method was used. This was due to the intent to present a method that can be used in a wide range of cases without having to meet any special requirements.

#### 2.3.1. Reference Model of the Porcelain Figurine

**Figure 11:**
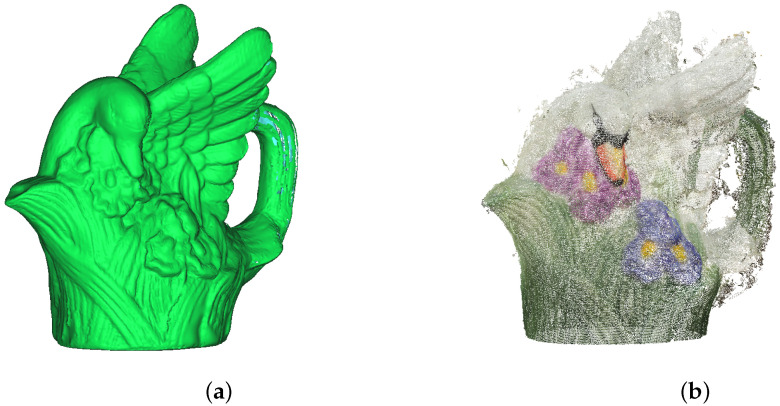
Porcelain figurine models: (**a**) mesh acquired by the 3D scanner, (**b**) point cloud obtained with the use of photogrammetric reconstruction.

The first testing datum was a little porcelain figurine, about 12 cm high. Its reference model was acquired with the use of a 3D scanner that operates in the field of structural light (Figure 11a). The scanner, unfortunately, was not able to reach all the covered parts of the object. The fragments of the figurine that did not have a correct digital representation were filtered out in the process of analysis. They were also the same fragments for which obtaining a correct representation during the photogrammetric reconstruction met with failure.

#### 2.3.2. Reference Model of the Castle in Nowy Wiśnicz

**Figure 12:**
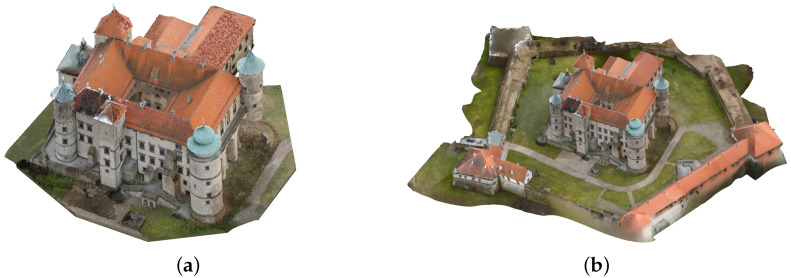
Polygonal models of the castle in Nowy Wiśnicz: (**a**) building only (4,133,352 faces), (**b**) the building with fortifications (1,966,487 faces).

The castle in Nowy Wiśnicz is in the north-eastern part of the town, less than 400 m from the Market Square. The origins of the castle date back to the fourteenth century, but its present shape is from the seventeenth century. It is a typical example of a palazzo in fortezza. Its quadrangular shape is accentuated by four corner towers. There is an additional segment to the south. The entire layout is surrounded by bastion fortifications in the shape of a pentagon with the longest dimension of 190 m. Together with bastions and curtain walls, it covers approx. 19,500 m^2^ (Figure 12b). The height of the castle itself, measured from the level of the courtyard inside the fortifications, is about 36 m (Figure 12a) [44].

Obtaining a reference model of such a large object was not possible using a 3D scanner. It was created using photogrammetric methods and verified via comparison with a point cloud obtained using airborne LiDAR. It is a combination of several partial models covering various parts of the castle. The individual partial models were obtained from separate sets of photos taken to ensure high quality. The photos were taken from human eye-level and from different heights, for which an UAV (Unmanned Aerial Vehicle) was used. There were 2300 photos in total. Partial reconstructions of the castle’s buildings were generated in Agisoft Metashape. They were then compiled using orthogonal versor matrix multiplication in Cloud Compare and combined into a single point cloud and then converted into a mesh model.

**Figure 13:**
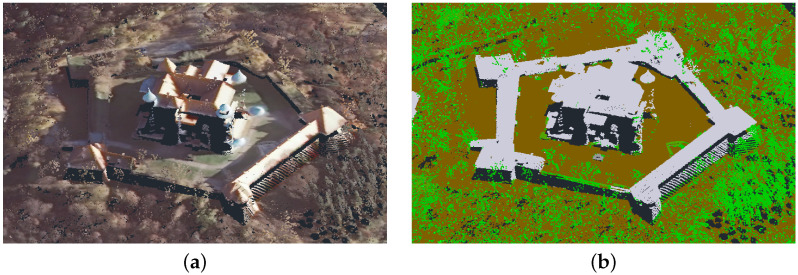
ISOK point cloud: scanning colors (**a**); class colors (**b**).

**Figure 14:**
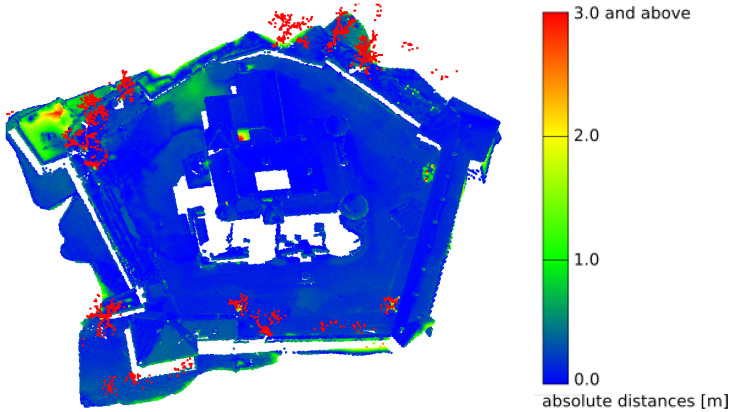
Distance error (RMS) for reconstructed model and reference LAS ISOK: LAS ISOK to reconstructed model—point-to-mesh mapping.

The point cloud used to validate the model represented a digital terrain and cover model. It was obtained from the state surveying repository. In Poland, by virtue of the law [45], the numerical terrain model and land cover data are currently available free of charge. They can be downloaded from the governmental servers of National Geoportal [46] as a grid of points with x, y, z coordinates, deployed at 1 m intervals. There are also LAS standard point cloud data available [47], acquired as a part of the ISOK project (National Land Cover IT System) [48]. As a result of this project, 98% of the territory of Poland was scanned, with a density of between 6–12 points/m^2^. The point cloud is available in the form of LAS files, where each point is represented by X, Y, Z, coordinates, RGB color (Figure 13a) and assigned to one of four classes: ground, structure, water and vegetation (Figure 13b) [47]. This enables the individual segmentation layers to be compared separately [49]. These data are reliable from a geolocation point of view, but insufficient for many engineering applications. Especially in the case of building walls, which, as elements with a mostly vertical geometry, are very poorly covered with points, as they are recorded via airborne LiDAR flyovers [50]. The fixed measurement interval does not provide the coordinates of distinctive elements of building geometry (corners, ridges, tops of towers). However, this is a feature that can be used for verification. The ISOK model points were used in this work for the systematic sampling of model correctness. The sparse ISOK point cloud precisely defines the space into which the dense cloud of the reference model must fit. After appropriate scaling and fitting of the reference model, the correctness was assessed by comparing the sparse ISOK cloud to the dense cloud of the reference model by examining the root mean square distance (RMS) between the superimposed structures (Figure 14). The RMS error estimation method allows the determination of a fitting error’s statistical values, which are expressed in spatial distance, which is well understood by engineers potentially using such models [51].

## 3. Results

The subject of our research was to determine whether the preprocessing of photos used to perform photogrammetric reconstruction affects its quality. Our research on grayscale images has shown that uniformity of pictures in terms of brightness and contrast can significantly improve the quality of a model obtained via reconstruction. However, using monochrome images has the disadvantage that the resulting texture does not precisely reflect the object’s appearance. For this reason, the photographs of the objects mentioned in the previous section were processed in color spaces in accordance with the methods presented.

The general scheme of the tests carried out on each set of photos is as follows:

1. Photo correction using one of the methods mentioned.
2. Creation of a sparse and then dense point cloud by photogrammetric reconstruction.
3. Registration using the ICP method to match the received data sets [52].
4. Comparison of individual reconstructions using the distance calculation: reconstructed model cloud to reference model cloud.
5. Statistical and visual analysis of the obtained results.

Classical statistical measures such as mean or standard deviation can be used when a variable is quantitative in nature. However, when the variable is ordinal, it is better to use positional measures such as median or interquartile range. In the case under study, the variable is the distance of a point derived from the photogrammetric reconstruction from the reference model obtained with the scanner. Therefore, the variable can be considered ordinal – the smaller the distance, the more correct the result. The analyzed statistical quantities are quartiles and in the interquartile range. Subsequent quartiles provide information about how far away from the original model the 25% ($Q_{1}$), 50% ($Q_{2}$—median), and 75% points ($Q_{3}$) are. These values are calculated from distances given in absolute (unsigned) quantities, so a distance of 0.10 cm is treated the same as a distance of −0.10 cm. However, in the case at hand, this is irrelevant because the objective is to determine the number of points that are within a given range of distances, without taking into account whether they are outside or inside the reference model. The smaller the value of a given quartile, the better the result because it means that a given percentage of points lies at a closer distance to the reference model. For example, in the case of the swan figure and the HE method in the L*a*b* space, the third quartile is 0.17 cm, which means that 75% of the points are closer to the reference model than this value. For the same method in RGB space, these 75% of the points are closer than 0.20 cm, which is a larger value, and therefore the points are further away from the reference model (Table 1).

Another measure that is calculated is the interquartile range (IQR), which is one of the values that determines dispersion. This measure is determined using a signed number and determines the degree of diversity—the higher the interquartile range value, the greater the variety of a feature. The value of this measure is calculated based on the difference between the third and first quartiles: $IQR=Q_{3}-Q_{1}$. In other words: the 50% of points are within the interval defined by the value of IQR. A narrower interval means a higher concentration of points in closer proximity to the reference model, so in the case under analysis, a lower value of IQR indicates a better result.

Based on quartile values obtained, it was also possible to perform an analysis of the length of the “tails”, i.e., the number of outlying points beyond a specific value of the distance from the reference model. The average of the values of $Q_{3}$ for all the measurements taken was assumed to be this particular value (σ). The values obtained determined how many points were at a significant distance from the reference model. These points can therefore be treated as incorrectly reconstructed. Due to the different number of points in the cloud for each reconstruction, what is important here is the value in percentage terms—the smaller it is, the fewer points are at a significant distance from the reference model, and thus it can be considered more accurate.

### 3.1. Case Study 1—The Porcelain Figurine

**Figure 15:**
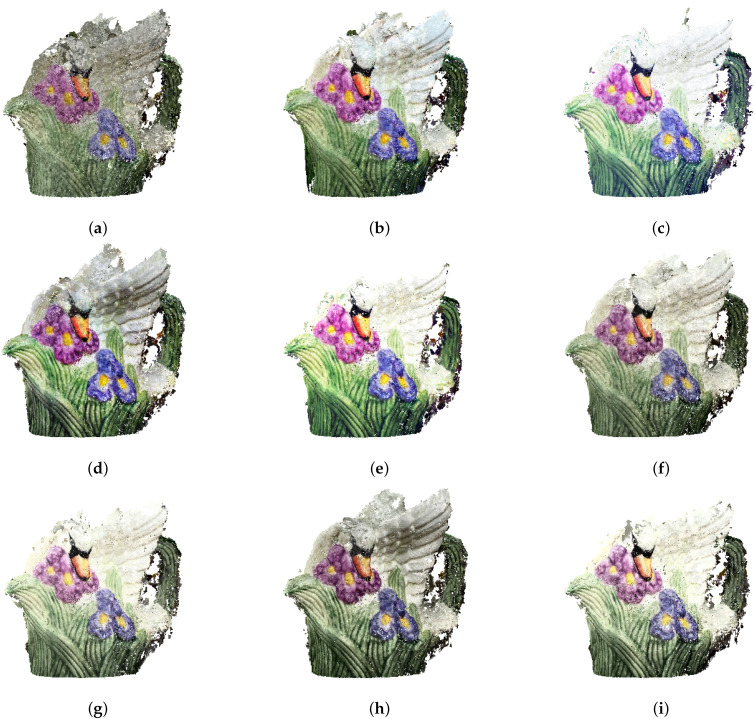
Dense point clouds of the porcelain figurine. Reconstructions based on photographs: (**a**) original; preprocessed in the RGB space with the use of: (**b**) histogram stretching, (**c**) histogram equalizing, (**d**) adaptive equalizing, (**e**) exact histogram matching; preprocessed in the L*a*b* space with the use of: (**f**) histogram stretching, (**g**) histogram equalizing, (**h**) adaptive equalizing, (**i**) exact histogram matching.

The set of images used for photogrammetry reconstruction consisted of 33 photographs. Twelve of them were underexposed, and eleven of them were overexposed. The dense clouds obtained in the figurine reconstruction process consisted of several hundred thousand points. These were then reviewed for duplicates, leading to the removal of approximately 10% of points from each cloud. The final number of obtained points is shown in Table 1. The obtained reconstructions are quite difficult to compare in terms of quality visually (Figure 15). However, the differences in contrast and color saturation of the textures are well demonstrated. Particularly notable is the low contrast in the reconstruction created using the original images (Figure 15a). As described in Section 2.2, the modification of the image in L*a*b* space involves transforming the histogram on the luminance channel without modifying the color channels, which can easily be seen in Figure 15f–i. On the other hand, modifying individual channels in the RGB color space results in changes in the saturation of individual colors (Figure 15b–e) and can also lead to errors in texture colors.

Upon a statistical analysis of the results (presented in Table 1), one can see very similar results for all investigated methods. The value of the first quartile in each case was 0.05 cm. The median values also differed very little. Only values for the third quartile showed a slightly bigger variation, where the best value was attained for the HE method in the L*a*b* space. However, it should be noted that the differences in individual values were minuscule and approximate the reconstruction obtained using unprocessed photograph.

The situation was slightly different when tail analysis was performed (Table 2). The σ value for the figurine was taken as 0.2 cm. This value was exceeded by 23.40% of points when reconstructed from images without enhancement, and only 17.84% of points when corrected using the HE method in L*a*b* space. In general, methods operating in L*a*b* space performed better in this case, with an average of 22.16% of points above the σ value, compared to 25.48% for methods operating in RGB space. A similar relationship can also be observed by analyzing the distribution of values for 2σ and 3σ. It should also be noted that the number of points located at a distance above 2σ, i.e., above 4 mm from the reference model, was relatively small—for the previously mentioned HE method in L*a*b* space, it is 3.62% of the total number of points in the cloud.

**Figure 16:**
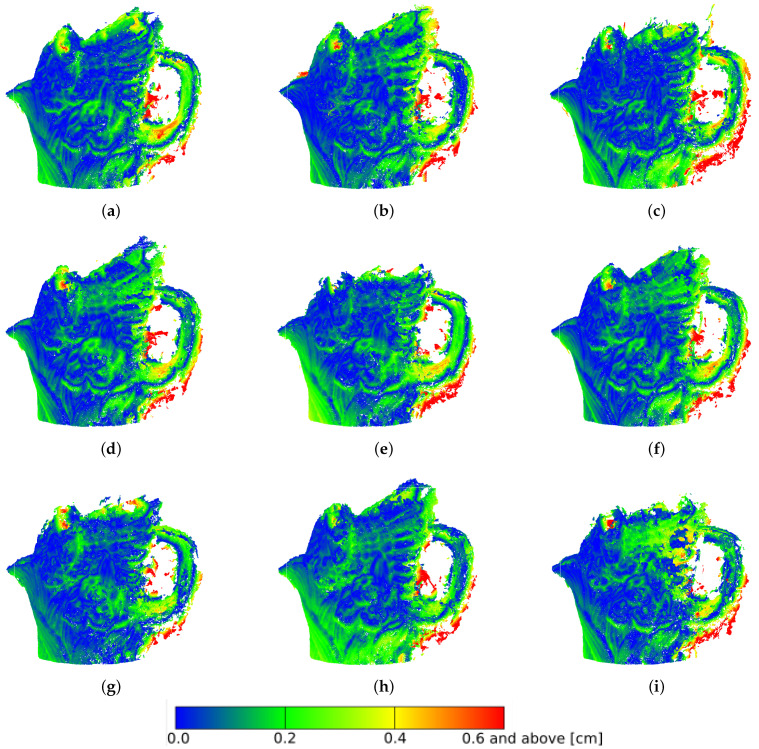
Dense point clouds of the porcelain figure. Distances to the model up to 3σ = 0.6 cm: (**a**) original; preprocessed in the RGB space with the use of: (**b**) histogram stretching, (**c**) histogram equalizing, (**d**) adaptive equalizing, (**e**) exact histogram matching; preprocessed in the L*a*b* space with the use of: (**f**) histogram stretching, (**g**) histogram equalizing, (**h**) adaptive equalizing, (**i**) exact histogram matching.

The distribution of deviations is not uniform over the entire surface of the model. The largest number is found in the ear region of the figure, as this section was the most difficult to reconstruct for the photogrammetric reconstruction algorithms (Figure 16). The different reconstructions were similar to each other in terms of error deviations. Visually, the largest number of points, located at distances above 3σ, was found for the HE method in RGB space (Figure 16c), which is confirmed by the numerical values (Table 2). It is also notable that for the method based on the EHM algorithm, the upper part of the figure was not correctly reconstructed for both RGB and L*a*b* space processed photographs (Figure 16e,i).

As mentioned, both original and preprocessed reconstructions are quite similar. This may suggest that in the case of easily accessible objects of small size, repeating the acquisition of a series of photographs with more attention to lighting conditions and correct camera settings would prove a better reconstruction improvement method than source image processing. To verify this hypothesis, an additional analysis was performed with photographs that met the proper acquisition conditions.

**Figure 17:**
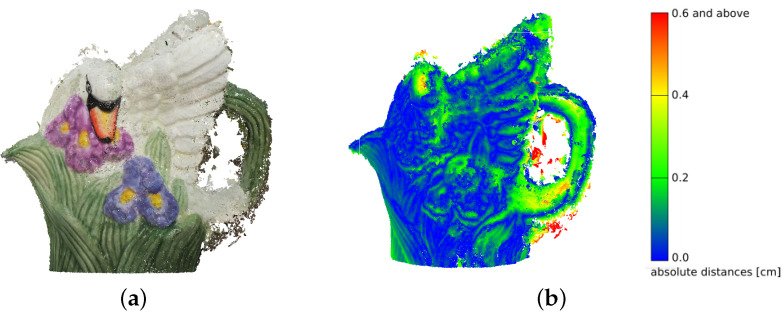
Improved reconstruction of the porcelain figurine: (**a**) RGB dense cloud, (**b**) absolute distance mapping.

Upon visual comparison of the reconstruction (Figure 17), it can be seen that the point cloud contained more detail than the others shown in Figure 15. Numerical data also confirmed this. The cloud consisted of 521,404 points, which was an increase of 39.7% relative to the reconstruction with the highest number of points (retrieved using the HS method in RGB space) and 48.9% more than the reconstruction made from the original images. However, the number of points in the cloud itself was not conclusive, as the points may not have been reconstructed correctly. Nevertheless, the repeated reconstruction gained some advantage here as well. Both quartiles and IQR values were 0.01 lower on average than the best reconstructions obtained by the histogram improvement method. The value of IQR, which is a measure of dispersion, was also 0.05 (21.7%) lower than the value obtained for the reconstructions made from the originally acquired images. The tail analysis also confirmed the desirability of re-taking the image sequence. The number of points above σ equaled 17.15%, with 23.40% for the original reconstruction and 17.84% for the best reconstruction obtained using the HE method in L*a*b* space. The number of points above 3σ represented only 0.46% of the total number of points, which was a decrease by 54.5% from the best reconstruction and 61.9% from the original one.

### 3.2. Case Study 2—The Castle in Nowy Wiśnicz

**Figure 18:**
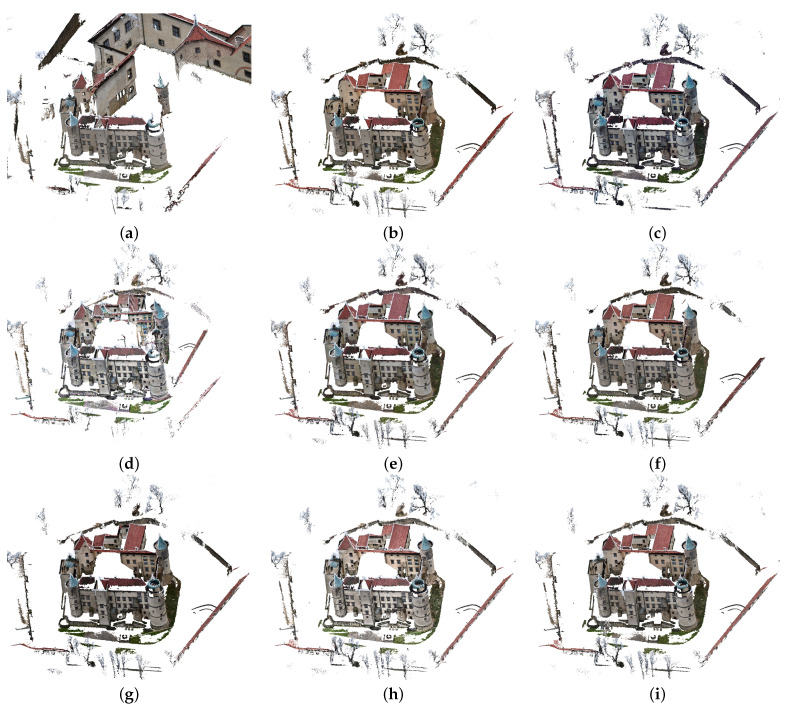
Dense point clouds of the castle. Reconstructions based on photographs: (**a**) original; preprocessed in the RGB space with the use of: (**b**) histogram stretching, (**c**) histogram equalizing, (**d**) adaptive equalizing, (**e**) exact histogram matching; preprocessed in the L*a*b* space with the use of: (**f**) histogram stretching, (**g**) histogram equalizing, (**h**) adaptive equalizing, (**i**) exact histogram matching.

**Figure 19:**
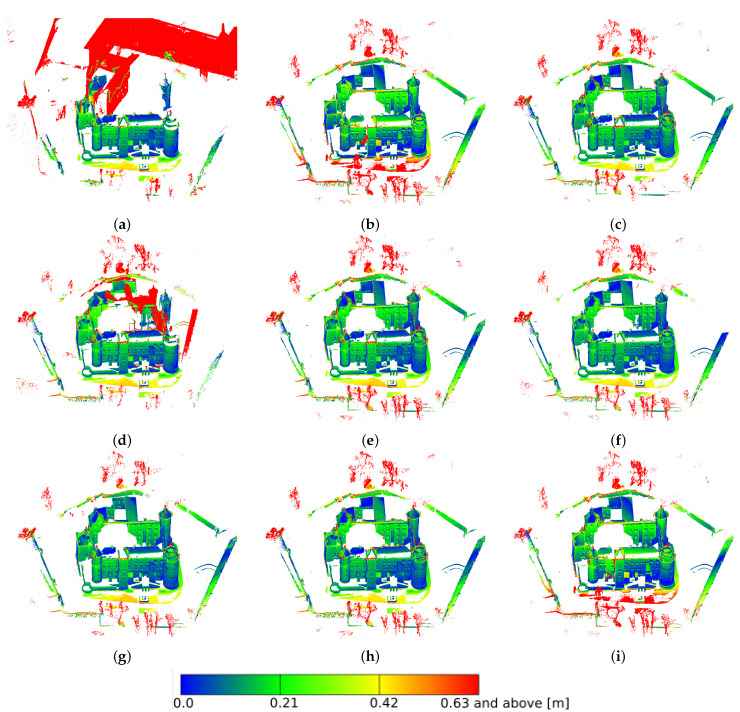
Dense point clouds of the castle. Distances to the model up to 3σ = 0.63 m: (**a**) original; preprocessed in the RGB space with the use of: (**b**) histogram stretching, (**c**) histogram equalizing, (**d**) adaptive equalizing, (**e**) exact histogram matching; preprocessed in the L*a*b* space with the use of: (**f**) histogram stretching, (**g**) histogram equalizing, (**h**) adaptive equalizing, (**i**) exact histogram matching.

The castle in Nowy Wiśnicz is an object several times larger than the presented model of the figurine. The number of images used in its reconstruction process was about ten times greater, and the number of points of the dense cloud about 100 times greater (about 30 million points—Table 3). The whole set of selected images used for photogrammetric reconstruction consisted of 338 photographs, and about 20% of them were underexposed. The first look at the resulting models allowed us to notice that the reconstruction made with the use of original photographs were incorrectly made (Figure 18a). In fact, this error inspired the research towards improving the quality of the reconstruction without repeating the photographic registration process. The defects could also be seen with the use of the AHE method in RGB space. The deviations were more clearly visible when analyzing images where the distance from the reference model was marked with the corresponding color (Figure 19). Similar to the reconstruction of the porcelain figurine, differences in contrast and color saturation of the textures were also apparent here. For reconstructions created using images processed in RGB space, color distortion was noticeable (Figure 18c–e). On the other hand, when using the AHE method in L*a*b* space, considerable texture brightening was visible.

Analysis of the statistical values strongly highlighted the defect in the original reconstruction. The value of $Q_{1}$ reached 11.78 m and IQR, which is a measure of dispersion as high as 75.90 m (Table 3). For reconstructions based on modified images, these values were several orders of magnitude smaller. The value of the first quartile oscillated around 5 cm, while the third quartile for almost all reconstructions was less than 20 cm. It was larger only in the previously mentioned AHE method in RGB space and amounted to 46 cm. This numerically confirmed the visually observed abnormality in this reconstruction. However, it should be noted that the $Q_{3}$ value was a small fraction (about 0.4%) of the castle dimensions, which demonstrates the correctness of the reconstruction since 75% of the points lied within this dimensional deviation from the reference model. It is interesting that each of the 338 photographs, was aligned by the software. This indicates that the reconstruction error occurs already at the keypoint extraction stage. This observation should be deeply investigated in subsequent studies.

The above conclusions are also confirmed by tail analysis (Table 4). The σ value was assumed to be 0.21 cm in this case. Almost all (89.22%) points for the reconstruction performed from unenhanced images were above this value. The lowest and therefore best values were achieved for the HS and HE enhancement methods in L*a*b* space—14.58% and 14.96%, respectively. Again, methods operating in L*a*b* space tended to perform better—on average, 16.47% of points were above the σ value, compared to 23.32% for the methods operating in RGB space. Of course, this was affected by the aforementioned reconstruction incorrectness for the AHE method in RGB, for which the σ value was 39.47%. The number of significant deviations from the reference model was the smallest for the methods operating in L*a*b* space. A deviation of more than 63 cm (3σ) was observed for less than 1% of the points for the three methods operating in this space. Among the methods operating in RGB, only in two cases, a deviation above 3σ was noted for about 1% of the points, while for the AHE method in this space, it was more than 23% of the points.

The fragments of the reconstruction with the largest deviation from the reference model were marked in Figure 19 in red. The reconstruction defect for the original images is clearly visible (Figure 19a) as is the one for the AHE method in RGB (Figure 19d), where additional elements of the castle towers and walls, positioned at the wrong angle, are visible. In all cases, the deviations occurred within the courtyard in front of the main entrance to the castle. In two cases, RGB HS and L*a*b* EHM, they were slightly larger (Figure 19b,i). An indication of this was also reflected in the statistics featured in Table 4. It is also noteworthy that a significant amount of the points that diverged the most from the reference model were associated with vegetation that was not present on the reference. Removing this vegetation would require manual manipulation of the reconstruction, which the authors wanted to avoid in order not to distort the results of the comparisons. Other inaccuracies included roof edges that were difficult to reconstruct. The least amount of such inaccuracies was found in the HS improvement in L*a*b* space (Figure 19f).

## 4. Discussion and Conclusions

The research presented in this paper was intended to verify the effectiveness of image enhancement methods for photogrammetric reconstruction. It is an extension of earlier studies performed on grayscale reduced images, which demonstrated that modifying the histogram of individual images can significantly improve reconstruction quality. However, monochromatic images do not fully represent the reconstructed object correctly due to the lack of realistic color reproduction. For this reason, we explored the performance of image modifications using histogram enhancement methods (HS, HE, AHE, EHM) in color spaces (RGB and L*a*b*). As demonstrated, histogram modifications affected the final shape of the reconstruction. The clearest example of this is the case study presented in Section 3.2, regarding the castle in Nowy Wiśnicz. Although the reconstruction based on the original photographs was incorrect, the other reconstructions, obtained using the modified photographs, were already accurate, which should be considered a success of the presented method. This is evident both from the visual side (Figure 18) and from the statistical analyses (Table 3 and Table 4). Each of the reconstructions looked slightly different, which also proves that modifications of the source photographs impacted the final result.

Two case studies were selected so as to present the method’s application on objects of different sizes. For a small object, the influence on the quality of the reconstruction was more visually noticeable. Statistically, the deviations of the reconstruction from the reference model were rather small, and it is difficult to unambiguously indicate which of the point clouds obtained represent better quality. However, this is influenced by the fact that the reconstruction performed based on original images was roughly correct, i.e., it reflected the object’s original shape. The reconstruction obtained from photographs taken with attention to appropriate lighting conditions indicates that proper image acquisition is advantageous over image preprocessing methods. However, registration cannot always be repeated for various reasons, such as the temporary availability of the object to be reconstructed. In such cases, improving the quality of the photographs using appropriate methods can give the expected results.

Architectural objects, such as the castle in Nowy Wiśnicz presented in Section 2.3.2, are significantly more challenging in terms of obtaining the correct exposure of the collected material. It is impossible to choose the optimal lighting by oneself, as it is directly related to the current weather conditions. Additionally, in the case of such an object, the number of photos that must be taken is much greater, which results in longer recording and data post-processing times. In most situations, it is not possible to verify the correctness of registration at the place of acquisition, e.g., by performing preliminary reconstruction. In such cases, it is reasonable to use image preprocessing, as demonstrated in this paper.

Based on the histogram correction methods analyzed, several conclusions can be drawn. Visual analyses of the obtained reconstructions (Figure 15 and Figure 18) indicated the superiority of methods that operate in L*a*b* space. In the RGB space, all the color channels are modified, which leads to color distortions in the textures. Similarly, from a statistical point of view, methods that operate in the L*a*b* space are more efficient. It is possible that this is related to the aforementioned falsification of the colors of the photographs, but such a conclusion requires further research. Our experiments did not result in any unequivocal recommendations regarding the superiority of specific histogram methods; however promising results were obtained using histogram equalization and histogram stretching in L*a*b* space. This is important since these methods are well-known and considered basic, are mathematically simple and do not require significant computation power. However, further research in this direction is required.

## Figures and Tables

**Table 1:** Number of points and statistical measures for the reconstruction of the porcelain figurine.

	Method	Aligned Cams	Points	Q1	Median	Q3	IQR
	original	24	350,232	0.05	0.11	0.19	0.23
L*a*b*	AHE	23	365,903	0.05	0.12	0.20	0.23
	EHM	23	300,097	0.05	0.10	0.19	0.19
	HE	24	313,622	0.05	0.10	0.17	0.20
	HS	27	338,858	0.05	0.11	0.19	0.21
RGB	AHE	23	337,649	0.05	0.11	0.19	0.21
	EHM	23	280,334	0.05	0.11	0.20	0.21
	HE	23	316,222	0.05	0.11	0.20	0.20
	HS	27	373,312	0.05	0.12	0.22	0.23
					avg:	0.20	

**Table 2:** Tail analysis for swan figure (σ=Q3=0.2).

	Method	σ	[%]	2σ	[%]	3σ	[%]
	original	81,949	23.40	16,829	4.81	4232	1.21
L*a*b*	AHE	92,521	25.29	12,898	3.52	4756	1.30
	EHM	69,061	23.01	14,381	4.79	3695	1.23
	HE	55,938	17.84	11,367	3.62	3165	1.01
	HS	76,199	22.49	13,910	4.10	3846	1.13
RGB	AHE	78,988	23.39	15,156	4.49	4268	1.26
	EHM	69,099	24.65	13,064	4.66	4015	1.43
	HE	79,759	25.22	18,559	5.87	6565	2.08
	HS	106,967	28.65	26,207	7.02	4564	1.22

**Table 3:** Number of points and statistical measures for the reconstruction of the castle in Nowy Wisnicz.

	Method	Aligned Cams	Points	Q1	Median	Q3	IQR
	original	338	41,833,893	11.78	47.73	87.30	75.90
L*a*b*	AHE	338	30,328,057	0.05	0.10	0.17	0.19
	EHM	338	31,144,627	0.04	0.10	0.19	0.20
	HE	338	28,241,790	0.05	0.10	0.17	0.19
	HS	338	29,980,758	0.04	0.10	0.16	0.18
RGB	AHE	338	30,774,070	0.06	0.16	0.46	0.31
	EHM	338	30,223,244	0.05	0.10	0.17	0.19
	HE	338	30,163,377	0.05	0.11	0.17	0.19
	HS	338	31,055,419	0.05	0.11	0.19	0.20
					avg:	0.21	

**Table 4:** Tail analysis (σ=Q3=0.21) for the reconstruction of the castle in Nowy Wisnicz.

	Method	σ	[%]	2σ	[%]	3σ	[%]
	original	37,325,857	89.22	36,496,517	87.24	36,157,816	86.43
L*a*b*	AHE	4,706,784	15.52	759,082	2.50	254,641	0.84
	EHM	6,477,239	20.80	1,889,706	6.07	736,932	2.37
	HE	4,225,890	14.96	661,988	2.34	159,952	0.57
	HS	4,372,294	14.58	764,709	2.55	258,619	0.86
RGB	AHE	12,145,976	39.47	8,087,475	26,28	7,209,241	23.43
	EHM	4,945,613	16.36	975,302	3.23	323,573	1.07
	HE	4,659,034	15.45	799,797	2.65	261,054	0.87
	HS	6,829,555	21.99	2,295,160	7.39	987,314	3.18

## Data Availability

The study used data in LAS format, which is freely available at https://mapy.geoportal.gov.pl/imap/Imgp_2.html (accessed on 25 March 2021).

## Abbreviations

The following abbreviations are used in this manuscript:

HS	histogram stretching
HE	histogram equalizing
AHE	adaptive histogram equalizing
EHM	exact histogram matching
CLAHE	contrast-limited adaptive histogram equalization
UAV	Unmanned Aerial Vehicle
LiDAR	Light Detection and Ranging
ISOK	IT System of the Country Protection
RMS	Root Mean Square
ICP	Iterative Closest Point
IQR	interquartile range
